# Lipoprotein(a), Immune Cells and Cardiovascular Outcomes in Patients with Premature Coronary Heart Disease

**DOI:** 10.3390/jpm12020269

**Published:** 2022-02-12

**Authors:** Olga I. Afanasieva, Alexandra V. Tyurina, Elena A. Klesareva, Tatiana I. Arefieva, Marat V. Ezhov, Sergei N. Pokrovsky

**Affiliations:** 1 Institute of Experimental Cardiology, National Medical Research Center of Cardiology, Ministry of Health of the Russian Federation, 121552 Moscow, Russia; afanasieva.cardio@yandex.ru (O.I.A.); hea@mail.ru (E.A.K.); tiarefieva@cardio.ru (T.I.A.); dr.pokrovsky@mail.ru (S.N.P.); 2 A.L. Myasnikov Institute of Clinical Cardiology, National Medical Research Center of Cardiology, Ministry of Health of the Russian Federation, 121552 Moscow, Russia; marat_ezhov@mail.ru

**Keywords:** lipoprotein(a), immune cells blood count, coronary heart disease

## Abstract

The detection of lipoprotein(a) [Lp(a)] in the artery wall at the stage of lipid-bands formation may indicate that it participates in the atherosclerosis local nonspecific inflammatory process. Innate immune cells are involved in atherogenesis, with monocytes playing a major role in the initiation of atherosclerosis, while neutrophils can contribute to plaque destabilization. This work studies the relationship between Lp(a), immune blood cells and major adverse cardiovascular events (MACE) in patients with the early manifestation of coronary heart disease (CHD). The study included 200 patients with chronic CHD, manifested up to the age of 55 in men and 60 in women. An increased Lp(a) concentration [hyperLp(a)] was shown to predict cardiovascular events in patients with premature CHD with long-term follow-up. According to the logistic regression analysis results, an increase in the monocyte count with OR = 4.58 (95% CI 1.04–20.06) or lymphocyte-to-monocyte ratio with OR = 0.82 (0.68–0.99), (*p* < 0.05 for both) was associated with MACE in patients with early CHD, regardless of gender, age, classical risk factors, atherogenic lipoproteins concentration and statin intake. The combination of an increased monocyte count and hyperLp(a) significantly increased the proportion of patients with early CHD with subsequent development of MACE (*p* = 0.02, *p*_trend_ = 0.003). The odds of cardiovascular events in patients with early CHD manifestation were highest in patients with an elevated lymphocyte-to-monocyte ratio and an elevated Lp(a) level. A higher neutrophil blood count and an elevated neutrophil-to-lymphocyte ratio determined the faster development of MACE in patients with a high Lp(a) concentration. The data obtained in this study suggest that the high atherothrombogenicity of Lp(a) is associated with the “inflammatory” component and the innate immune cells involvement in this process. Thus, the easily calculated immunological ratios of blood cells and Lp(a) concentrations can be considered simple predictors of future cardiovascular events.

## 1. Introduction

Atherosclerotic cardiovascular diseases (ASCVDs) have remained the leading cause of death worldwide over the past 15 years, despite continued advances in pharmaceuticals and technology [1]. Although there is some progress in the treatment of cardiovascular disease, a trend towards the earlier development of ASCVD and associated cardiovascular events can be observed [2,3,4,5]. А significant proportion of major adverse cardiovascular events (MACE) could be avoided via the correction of modifiable risk factors [6], but despite this, the residual risk of CVD development remains [7].

Signs of the local nonspecific inflammatory process in atherosclerosis are traced from the earliest stages of the vessel-wall-lesion development to the stage of destabilization and atherosclerotic plaque damage [8,9]. There is a growing body of evidence supporting the idea that lipoprotein(a) [Lp(a)] and inflammation are important factors in residual risk of ASCVD development [10]. The structural organization of Lp(a), the genetic nature of Lp(a) levels inheritance and its presence in the walls of the arteries already at the stage of lipid-band formation suggested it can participate in this inflammatory process. Several types of immune cells are involved in the atherosclerotic process, such as monocytes, T and B lymphocytes, neutrophils. The results of some studies have shown that enhanced myelopoiesis plays a central role in increasing monocyte and neutrophil numbers in cardiovascular disease and intensifies the formation of atherosclerotic lesions [11]. Despite exploring Lp(a) for several decades, many aspects regarding its role in the development of atherosclerosis are still unclear. There are data that Lp(a) is able to enhance the production of inflammatory monocytes in the bone marrow [12]. We have recently shown that in patients with an elevated Lp(a) level, the absolute and relative content of non-classical CD14+CD16++ monocytes is significantly higher [13], indicating the ability of Lp(a) to influence monocytes. Residual cardiovascular risk in patients with atherosclerosis, despite adequate hypolipidemic therapy, could be related to the increased concentration of Lp(a) and, therefore, the possible relationship between Lp(a) and the vascular wall inflammation deserves further evaluation. Thus, the aim of this study is to investigate the association between Lp(a) concentration, immune blood cells count, and cardiovascular outcomes in patients with early manifestation of coronary heart disease (CHD).

## 2. Materials and Methods

**Figure A1:**
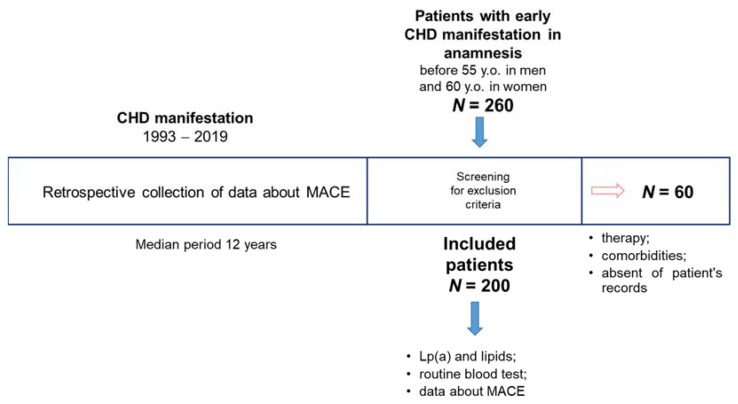
Design of the study. 260 patients with an early history of CHD hospitalized and screened between 2019 and 2021 were included during the screening phase. 60 people were expelled according to the exclusion criteria. The information about the presence or absence of any significant cardiovascular events as well as the date of each such event from the CHD manifestation was available for all patients according to medical records. All patients, after signing an informed consent, were re-examined, relevant medical data were collected, biochemical tests and routine blood tests were performed.

This retrospective study included consecutive patients who underwent a repeat examination at the National Medical Research Center of Cardiology of the Ministry of Health of the Russian Federation between 2019 and 2021 (Figure A1). The study was carried out in accordance with Good Clinical Practice and the principles of the Declaration of Helsinki. The study protocol was approved by the local ethics committee and written informed consent was obtained from all the patients before their enrollment. We included 200 patients aged 59 ± 9 years with a history of early CHD manifestation (before 55 and 60 years in men and women, respectively). Clinical charts were available from the time of manifestation of CHD for all the included patients. Exclusion criteria were severe comorbidities affecting the prognosis; dementia; alcohol abuse; autoimmune and infectious diseases; and treatment with any hormones, PCSK9 inhibitors, and apheresis. All the patients were examined and interviewed to evaluate the course of the disease and the presence of classical atherosclerosis risk factors. Arterial hypertension was diagnosed if the patient took antihypertensives, or in cases of of systolic blood pressure level being above 140 mmHg and/or diastolic blood pressure being above 90 mmHg according to two blood-pressure measurements on two different visits. Smoking status was assessed as never smoker, former smoker or current smoker [14]. Type 2 diabetes was diagnosed according to the World Health Organization criteria [15,16]. Body mass index (BMI) was calculated for all the participants and obesity was recorded at BMI ≥ 30 kg/m^2^.

The lipids (total cholesterol (ТС), triglyceride (TG) and high-density lipoprotein cholesterol (HDL-C)) were determined by enzymatic colorimetric method on Hitachi 912 biochemical analyzers (Roche Diagnostics, Basal, Switzerland) and Architect C-8000 (Abbott, Abbott Park, Illinois, USA). The quality control of the studies was accomplished with the control sera Precinorm and Precipat (Roche Diagnostics, Basal, Switzerland). The low-density lipoprotein cholesterol (LDL-C) level was calculated with Martin–Hopkins’s formula:

(1) $$\mathrm{LDL}–C=\;\mathrm{TC}\;–\;\mathrm{HDL}–C-\frac{\mathrm{TG}}{f},$$

where f is an adjustable factor from 3.1 to 11.9 (result in mg/dL).

The concentration of LDL-C corrected for Lp(a)-cholesterol (LDL-C corr.) was calculated by Dahlen modification [17]:

(2) $$\mathrm{LDL}–C\;\mathrm{corr}\;=\;\mathrm{LDL}–C–\;0.3\times \frac{\mathrm{Lp}\left(a\right)}{38.7},\;(\mathrm{result}\;\mathrm{in}\;\mathrm{mmol}/L),$$

Lp(a) concentration was determined using an enzyme immunoassay with sheep monospecific polyclonal antibodies against human Lp(a), as previously described [18]. The sensitivity of the method was 0.2 mg/dL, the intra-plate and between experiments variation coefficients were 3.8% and 9.8% in the Lp(a) concentration ranged from 5 to 190 mg/dL. The method was validated with two kits, TintElize Lp(a) (Biopool AB, Umea, Sweden) and Immunozym Lp(a) (Progen Biotechnik GmbH, Heidelberg, Germany). The control serum (Technoclone, Vienna, Austria) was approved by the International Federation of Clinical Chemistry and was used to standardize the ELISA.

In addition, a routine blood test with the absolute count of leukocytes, the absolute and relative counts of lymphocytes, neutrophils, and monocytes was performed in all the patients. The lymphocyte-to-monocyte ratio (LMR) and neutrophil-to-lymphocyte ratio (NLR) were calculated as the ratios of the absolute count of lymphocyte to absolute count of monocyte and absolute count of neutrophil to absolute count of lymphocyte.

A statistical analysis was performed using a MedCalc 20.022. (MedCalc Software Ltd., Ostend, Belgium). The results were presented as a mean value ± standard deviation or median with 25th and 75th percentiles for normal or abnormal distribution according to Kolmogorov–Smirnov test, respectively. Student’s parametric t-test and non-parametric Mann–Whitney test were used when comparing the quantitative data of the two groups. Fischer’s exact test was used to estimate frequency data between groups. The Spearman rank correlation and multiple regression or logistic analysis were used. Odds ratio (OR) and 95% confidence intervals (CI) were calculated to evaluate associations between outcomes and study parameters. ROC-analysis was conducted to determine the cut-off criterion of Lp(a) level or lymphocyte-to-monocyte or neutrophil-to-lymphocyte ratios and associated sensitivity and specificity for MACE. Apart from that, and the Kaplan–Meier survival curve was analyzed because parameters such as sex, age of CHD manifestation, statin medication, and hyperLp(a) (≥30 mg/dL), remained unchanged during the whole time from CHD manifestation until now.

## 3. Results

Most of the patients were males (*n* = 166, 83%). Arterial hypertension was observed in 87% of the patients, type 2 diabetes in 30%, and 63% were current or former smokers. All the patients took statins at an adequate dose, the average dose equivalent to atorvastatin was 43.4 ± 21.3 mg. Another therapy is presented in the Table A1.

Over the median follow-up of 12 years, starting from the time of CHD manifestation in 121 out of 200 patients, the following major adverse cardiovascular events (MACE) were distinguished: non-fatal myocardial infarction (*n* = 57, 29%), coronary artery bypass grafting (*n* = 65, 33%), hospitalizations for unstable angina (*n* = 35, 18%), and ischemic stroke (*n* = 14, 7%).

There were no differences in terms of age, gender, classical risk factors frequency, baseline levels of TC, TG, HDL-C or LDL-C, hypolipidemic and antiplatelet therapy in the studied groups (Table 1).

**Figure 1:**
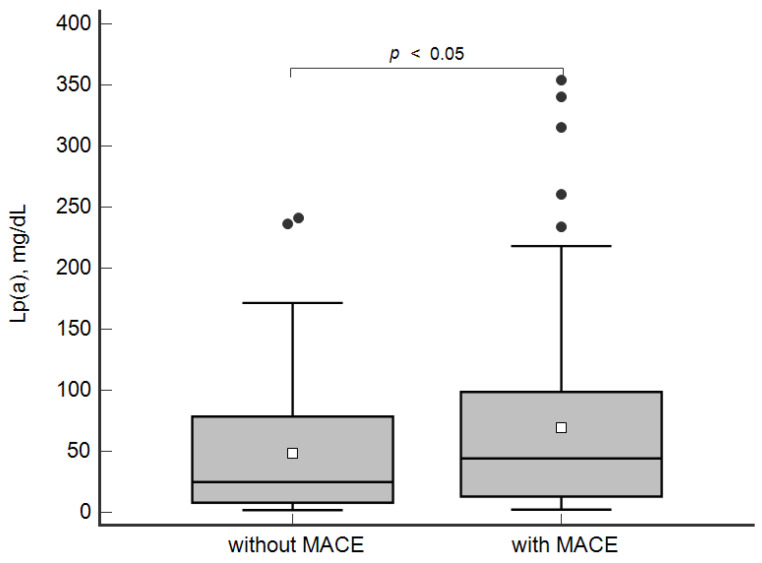
Concentration of lipoprotein(a) in patients with and without MACE. Data are presented as Box-and-Whisker plot: a grey box is drawn from the 25% and 75%; a horizontal line is a median (50%), a white square symbol is a mean, black points are values to the above of 1.5 × IQR (the Interquartile range (IQR) is calculated as 75%–25%).

The concentration of Lp(a) was higher in the patients with MACE in comparison with the group without MACE: 44 [13; 98] mg/dL and 25 [8; 79] mg/dL, respectively, *p* < 0.05 (Figure 1).

According to ROC-analysis, the concentration of Lp(a) ≥ 30 mg/dL was associated with MACE with 60% sensitivity and 54% specificity (area under the curve 0.59; 95% CI 0.51–0.65; *p* < 0.05). The ROC analysis for the two cut-off points—30 and 50 mg/dL—showed no significant differences, but the AUC was slightly higher for the cut-off point 30 mg/dL vs. 50 mg/dL: 0.57; 0.50–0.64 vs. 0.56; 0.49–0.63, *p* > 0.05.

**Figure 2:**
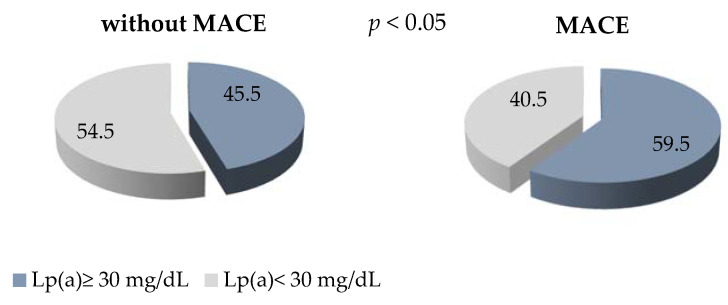
The proportion of patients with Lp (a) ≥ 30 mg/dL in groups with and without MACE.

The level of Lp(a) ≥ 30 mg/dl detected in 60% of the patients with early manifestation of CHD and MACE in comparison with 45% of the patients without MACE is presented, with *p* < 0.05 (Figure 2). The OR of MACE in patients with Lp(a) concentration of ≥ 30 mg/dL was 1.25 (0.99–1.58), *p* = 0.06.

**Figure A2:**
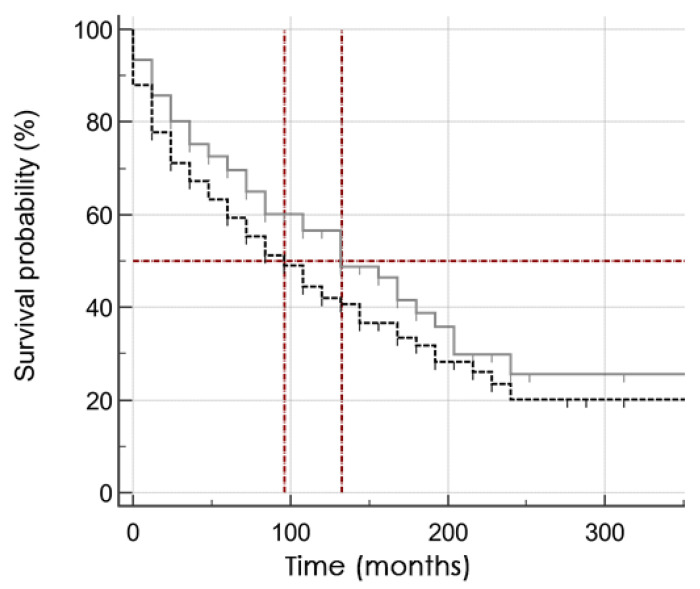
Kaplan–Meier survival curve in subgroups of patients Lp(a) < 30 mg/dL (grey solid line) and Lp(a) ≥ 30 mg/dL (black dotted line). 50% of patients in the group with elevated Lp(a) concentration lived to MACE after 96 (95% CI 60–144) months vs. 132 months (95% CI 84–192).

A trend towards a more rapid development of MACE was detected in the patients with Lp(a) ≥ 30 mg/dL. The patients without MACE but with Lp(a) ≥ 30 mg/dL had a mean survival time of 143 ± 15 months versus 168 ± 18 months in those with Lp(a) < 30 mg/dL, *p* < 0.01 (Figure A2).

The blood-cells count did not significantly differ in the patients with and without MACE (Table 2).

A correlation analysis showed no significant associations between immune-blood cells count and Lp(a) concentrations or other atherogenic lipoproteins.

According to the logistic regression analysis, an increase in Lp(a) concentration by 10 mg/dL with OR = 1.06 (95% CI 1.00–1.14), age increase by one year with OR = 1.04 (1.00–1.08) and male sex of the patients with OR = 2.57 (1.07–6.19) were independently associated with MACE, regardless of other risk factors, lipids, and the monocytes count.

**Figure 3:**
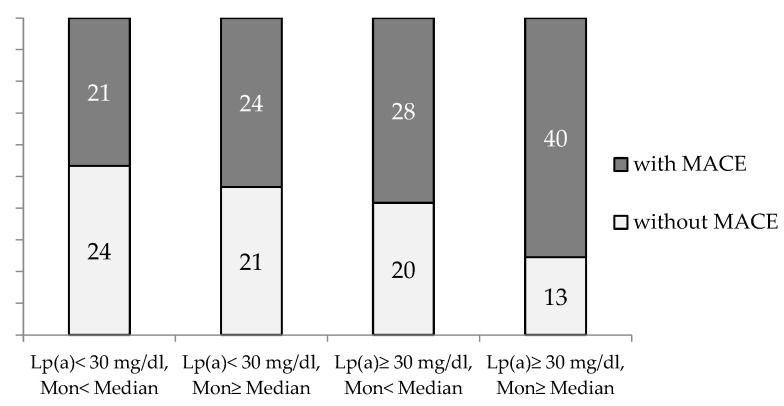
The proportion of CHD patients with and without MACE during observation period depends on blood monocyte count and Lp(a) concentration. Mon—monocytes. Median for Mon = 0.54 × 10^9^/L.

The combination of an elevated monocyte count (above median) in the presence of Lp(a) ≥ 30 mg/dL significantly increased the proportion of the patients with MACE (*p* = 0.02, *p*_trend_ = 0.003) (Figure 3).

The lymphocyte-to-monocyte ratio was lower in the group of the patients with MACE during the observation period 3.9 ± 2.5 vs. 4.6 ± 1.9, *p* = 0.05. According to the ROC analysis, the lymphocyte-to-monocyte ratio was significantly associated with MACE (AUC = 0.61, *p* = 0.01) and the cut-off value was calculated as <4.56 (a sensitivity of 69% and a specificity 49%). The median of the distribution for the lymphocyte-to-monocyte ratio was 4.18. This value discriminates MACE with a sensitivity of 60% and a specificity of 55% and was chosen as the most suitable for the proceeding calculations.

**Figure 4:**
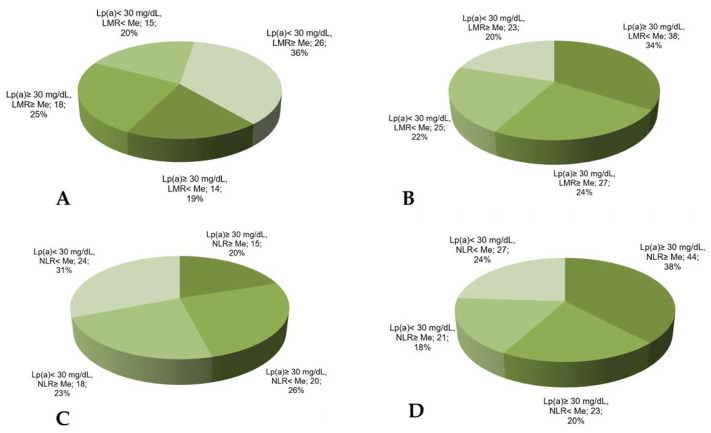
The proportion of CHD patients according to Lp(a) concentration, lymphocyte-to-monocyte ratio, or neutrophil-to-lymphocyte ratio in groups without (**A**,**B**) or with (**C**,**D**) MACE. LMR—lymphocyte-to-monocyte ratio, NLR—neutrophil-to-lymphocyte ratio, Me—median. Median for LMR = 4.18, for NLR = 2.66.

The combination of the reduced lymphocyte-to-monocyte ratio with Lp(a) ≥ 30 mg/dL was found in 34% of the patients with MACE vs. 19% in the patients without MACE (Figure 4) and significantly increased the chance of developing MACE in such patients (Table 3).

The neutrophil-to-lymphocyte ratio has no significant association with MACE (AUC = 0.56, *p* = 0.07) according to the ROC analysis. Nevertheless, the proportion of the patients with Lp(a) ≥ 30 mg/dL and the neutrophil-to-lymphocyte ratio above the median was maximal in the patients with MACE (Figure 4).

According to the logistic-regression analysis, an increase in the lymphocyte-to-monocyte ratio per unit was associated with MACE in the patients with the early CHD manifestation regardless of their sex, age, risk factors (arterial hypertension, diabetes, smoking), lipid levels, and statin intake (OR = 0.8 (0.7–1.0), *p* = 0.04). The neutrophils count and neutrophil-to-lymphocyte ratio were not associated with MACE in the same model.

**Figure 5:**
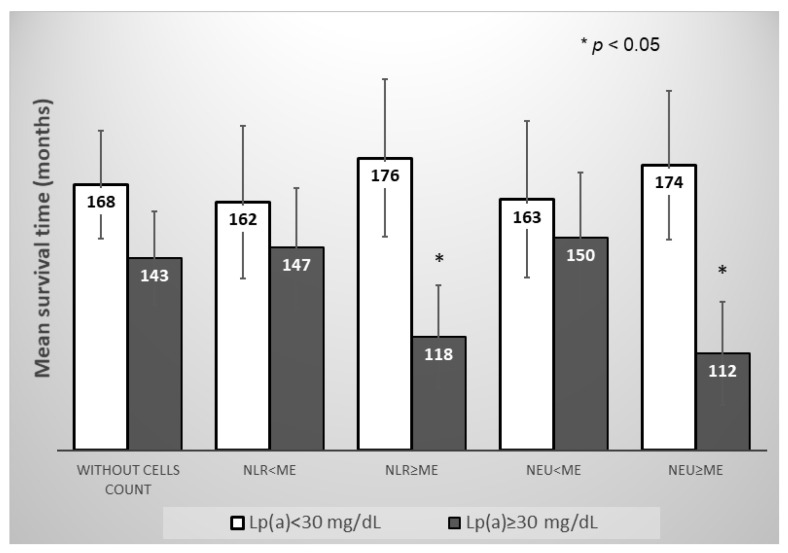
Mean survival time without MACE in subgroups of patients depending on the of Lp(a) concentration, the neutrophils blood count, or the neutrophil-to-lymphocyte ratio. NLR—neutrophil-to-lymphocyte ratio, NEU—neutrophil, Me—median. Median for NLR = 2.66, for Neu = 5.04 × 10^9^/L.

**Figure 6:**
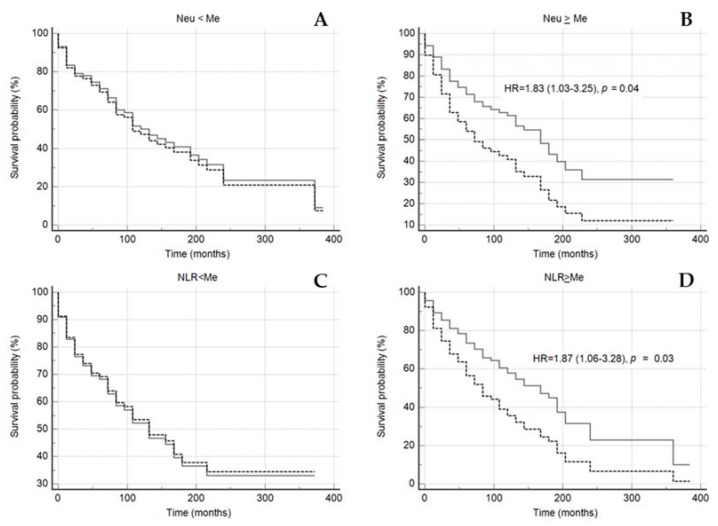
Kaplan–Meier survival without events curves in subgroups of patients with neutrophils count < median (**A**) or ≥ median (**B**), NLR < median (**C**) or ≥ median (**D**) depending on the of Lp(a) < 30 mg/dL (grey solid line) and Lp(a) ≥ 30 mg/dL (black dotted line). NLR—neutrophil-to-lymphocyte ratio, NEU—neutrophil, Me—median. Median for NLR 2.66, for Neu 5.04 × 10^9^/L. Sex, age of CHD manifestation, statin medication was included in model of Cox proportional regression.

However, in the subgroup of the patients with neutrophil-to-lymphocyte ratio and neutrophils count above the median, the Lp(a) concentration ≥ 30 mg/dL significantly reduced the mean survival time for MACE, in contrast to the subgroup of the patients with neutrophils count below the median (Figure 5 and Figure 6).

## 4. Discussion

The hypothesis that Lp(a) pathogenicity is associated with inflammation and that immune cells, and monocytes in particular, are involved, was expressed quite a long time ago [19]. The relationship between the innate immune system and Lp(a) is based on the fact that oxidized phospholipids localized on Lp(a) can be recognized by receptors of innate immunity [20].

Inflammation in the arterial vessel wall is detected in subjects with increased concentration of Lp(a). In contrast, monocytes isolated from the blood of patients with a high concentration of Lp(a) demonstrate an increased ability to transendothelial migration [21].

The activation of the innate immune system in patients with hyperlipoproteinemia(a) is consistent with our recent study, which demonstrated that in patients with an elevated Lp(a) concentration, there is a redistribution of monocyte subpopulations towards an increase in CD14+CD16++ monocytes [13]. Furthermore, the combination of hyperlipoproteinemia(a) and a higher content of intermediate monocytes was associated with a significant increase in stenotic plaques in all major coronary arteries.

The results of this retrospective study, in which patients with early CHD manifestation participated, showed that an increased monocyte count on the background of hyperlipoproteinemia(a) is associated with a 2.7-fold increase in the chance of MACE developing compared to those with Lp(a) < 30 mg/dl and the monocyte count below the median.

Experiments on apoE-/- mice demonstrated that myocardial infarction caused by artery ligation leads to an accelerated accumulation of innate immune cells, namely, an increase in both the total number of monocytes/macrophages in the aorta and a subpopulation of pro-inflammatory monocytes with a high content of Ly-6C as well as larger atherosclerotic lesions with a more advanced morphology relative to individuals without myocardial infarction [22]. The secreted pro-inflammatory cytokines by activated monocytes can accelerate the atherosclerotic process and contribute to the further destabilization of atherosclerotic plaques.

The neutrophil-to-lymphocyte ratio, as a possible marker of chronic systemic inflammation, is a simple predictor for both cardiovascular events and cardiovascular death in large prospective observational studies [23,24].

In our study, a neutrophil-to-lymphocyte ratio of more than 2.66 with an elevated Lp(a) concentration showed a twofold chance of MACE in patients with early CHD manifestation. A faster onset of MACE was demonstrated in patients with Lp(a) > 30 mg/dL and elevated neutrophil blood count or neutrophil-to-lymphocyte ratio. However, the correlation between Lp(a) concentration and neutrophils was not detected. Moreover, a correlation between HDL-C and neutrophil-to-lymphocyte ratio that has been described for healthy subjects, was not found in the patients with early manifestation of CHD [25].

Neutrophil-to-lymphocyte ratio as a marker of systemic inflammation has no specificity and is of prognostic value in oncology, sepsis, and other conditions [26,27]. However, a growing body of evidence demonstrates that neutrophils play an integral role in atherosclerotic cardiovascular disease development. The ability of neutrophils to enhance monocyte adhesion and transmigration to atherosclerotic plaques may contribute to a more severe atherosclerotic process in patients with an elevated Lp(a) level and early manifestation of CHD. There is evidence that neutrophils are localized near plaques that are prone to rupture [28]. Previously, it has been shown that the concentration of matrix metalloproteinase 9 was positively associated with the size of the necrotic core of atherosclerotic plaques and was inversely related to the fibrous tissue content of the plaques in patients with chronic CHD and Lp(a) level > 60 mg/dL [29]. The secretion of active proteases by neutrophils metalloproteinase 9, which degrades the fibrous capsule, may also explain the effects of neutrophils on MACE development in patients with elevated Lp(a) concentrations.

Despite the prominent role of both dyslipidemia and inflammation as crucial factors in atherogenesis [30], the diversity and sequence of inflammatory processes and the involvement of atherogenic lipoproteins are still unclear.

All the patients in the present study took statins, but most patients had MACE during a median of 12 years of follow-up, and the groups did not differ in terms of LDL-C concentration.

Patients with early CHD belong to the category of very high cardiovascular risk, which may explain the high percentage of cases with MACE during prolonged observation. In addition, in 78% of patients CHD has been manifested with myocardial infarction, which significantly worsens the prognosis. There are identical results for the high 15-year mortality in patients with early myocardial infarction in a long-term survival study [31]. Overall, 65% of patients had at least one serious cardiovascular event and/or death in the study Yagel and colleagues, examining cardiovascular outcomes in patients with early acute coronary syndrome [32]. More, than half (52.9%) of patients with early CHD had at least one cardiovascular event during 10 years of follow-up in another study of long-term prognosis [33]. The observation period in our study was more than 12 years for 50% and more than 18 years for 25% of included patients.

Male gender is a risk factor for cardiovascular diseases, especially for early CHD manifestation, and the number of men in our study was higher than the number of women. This corresponded with the prospective observational cohort study GENESIS-PROXY (Gender and Sex determinants of cardiovascular disease: from bench to beyond-Premature Acute Coronary Syndrome) [34] and another study devoted to the long-term prognosis in patients with early coronary heart disease [33]. In the study of risk profiles, sex-related differences, and outcomes in a contemporary population of young patients with CAD, men also made up 70% of the study group [5].

Although the existing effective methods of lipid-lowering therapy significantly reduce the burden of cardiovascular diseases, their effect on inflammation is contradictory. The results of a post hoc analysis of the FOURIER study showed that patients with a CRP level > 2 mg/L had a higher risk of MACE compared to participants with normal CRP levels, despite achieving the target LDL-C levels [35]. The risk associated with an increased Lp(a) concentration was also significantly higher in patients with more pronounced inflammation and higher CRP > 2 mg/L [36].

A reduction of cardiovascular events risk while taking anti-inflammatory drugs has been demonstrated in several clinical studies—Canakinumab Anti-inflammatory Thrombosis Outcome Study (CANTOS) that used therapeutic monoclonal antibodies against IL-1β (canakinumab), and LoDoCo2 study where low doses of colchicine in patients with chronic CHD were used [37,38].

We hypothesize a relationship between chronic inflammation processes, Lp(a) concentrations, and the development of MACE. The clinical significance of the present study results is related to the fact that patients with early CHD manifestation and elevated Lp(a) chronic inflammation, expressed as an increased ratio of immune blood cells, are associated with a more severe disease. The ratios could be calculated from a routine blood test, and their association with a prospective prognosis for various diseases has been shown above [24]. We propose assessing such ratios in patients with early CHD manifestation, especially in patients with elevated Lp(a) levels. Elevated levels of circulating monocytes and neutrophils in patients with early CHD manifestation and subsequent MACE can provide a pool of inflammatory cells available for recruitment into the growing arteries and their secreted pro-inflammatory cytokines and can accelerate the atherosclerotic process and thus, contribute to further destabilization of atherosclerotic plaques, leading to the subsequent MACE.

## 5. Study Limitation

Our study has some limitations. Patients had CHD manifestation in the past, prior to enrolling in the study (median 12 years). Currently, all the patients are taking statins at adequate doses, but we have no precise data on the regularity of intake from the beginning of the disease to the present time. Because our study was retrospective, we cannot say that the neutrophils count, and the neutrophil-to-lymphocyte ratio were elevated at CHD manifestation. However, we assumed the relative stability of neutrophil blood counts and neutrophil-to-lymphocyte ratio by dividing the patients into subgroups according to these parameters and analyzing survival curves [23].

The ELISA method for Lp(a) determination was sensitive to apolipoprotein(a) isoforms, resulting in a slight increase in the Lp(a) concentration in samples with high molecular weight apo(a) isoforms. The absolute bias (median [25%; 75%]) was ~1.5 [−0.4; 5.7] mg/dL. The high variability in the Lp(a) measurement, regardless of apo(a) isoforms, and the nonsignificant bias in the absolute Lp(a) concentration in our ELISA method makes it possible to assume that sensitivity of ELISA to apo(a) isoforms did not affect the results of the study. In addition, there were no significant differences in the Lp(a)-associated relative risk of CAD in studies using methods both sensitive and insensitive to the size of apo(a) isoforms, according to meta-analysis [39].

According to the results of a recent prospective ACCELERATE trial, the significant association between an elevated Lp(a) concentration and time to first MACE was found in patients with CRP levels ≥ 2 mg/L [36]. We did not determine interleukin or C-reactive protein concentrations, which is also a limitation of the study.

## 6. Conclusions

An elevated Lp(a) concentration was shown to be a predictor of cardiovascular events in patients with premature CHD with long-term follow-up. The likelihood of cardiovascular events in patients with early CHD manifestation were highest in patients with an elevated lymphocyte-to-monocyte ratio and an elevated Lp(a) level. A higher neutrophil blood count and an elevated neutrophil-to-lymphocyte ratio determine the faster development of MACE in patients with a high Lp(a) concentration. Thus, the easily calculated immunological ratios of blood cells and Lp(a) concentration can be considered as simple predictors of future cardiovascular events.

## Figures and Tables

**Table 1:** The patients’ characteristics depending on the occurrence of MACE.

	without MACE *n* = 79	MACE *n* = 121	*р*-Value
Men	61 (77%)	105 (87%)	0.1
Age, years	57.6 ± 8.5	59.5 ± 9.0	0.5
Age of CHD manifestation, years	47.7 ± 6.8	45.9 ± 7.9	0.2
BMI, kg/m^2^	29.4 ± 6.3	30.0 ± 5.0	0.5
Follow-up, years	10 ± 7	13 ± 8	0.4
Obesity	45 (57%)	57 (47%)	0.2
Arterial hypertension	70 (89%)	102 (84%)	0.6
Smoking	47 (60%)	79 (65%)	0.5
Family history of CVD	32 (41%)	39 (32%)	0.3
Type 2 diabetes	25 (32.6%)	35 (29%)	0.8
TC, mmol/L	4.3 ± 1.2	4.3 ± 1.1	1.0
TG, mmol/L	1.5 [1.1; 2.1]	1.5 [1.1; 2.2]	1.0
HDL-C, mmol/L	1.1 ± 0.3	1.2 ± 0.3	0.9
LDL-C, mmol/L	2.5 ± 1.1	2.4 ± 1.0	0.4
LDL-C corr, mmol/L	2.2 ± 1.1	1.8 ± 1.1	0.1
Average dose of statins, mg	42 ± 20	45 ± 22	0.3
LDL-C < 1.4 mmol/L	10 (13%)	14 (12%)	0.8
Antiplatelet/anticoagulant	65 (82%)	101 (83%)	1.0

Data are presented as mean ± standard deviation, or median [25%; 75%], or *n* (%).

**Table 2:** Blood cells counts in patients with and without MACE.

	without MACE *n* = 79	MACE *n* = 121	*р*-Value
Leukocytes, 10^9^/L	7.8 [6.6; 9.2]	7.7 [6.3; 8.9]	0.64
Lymphocytes, 10^9^/L	2.2 [1.7; 2.9]	2.0 [1.6; 2.5]	0.12
Lymphocytes, %	28.9 [24.4; 35.5]	27.5 [22.3; 33.2]	0.10
Neutrophils, 10^9^/L	4.7 [3.7; 5.5]	4.5 [3.8; 5.7]	0.85
Neutrophils, %	59.8 [53.8; 64.9]	61.8 [55.8; 67.7]	0.14
Monocytes, 10^9^/L	0.5 [0.4; 0.6]	0.6 [0.4; 0.7]	0.20
Monocytes, %	6.8 [5.4; 8.1]	7.0 [5.9; 9.2]	0.09
Basophiles, 10^9^/L	0.07 [0.05; 0.09]	0.06 [0.05; 0.09]	0.50
Basophils, %	0.90 [0.68; 1.16]	0.87 [0.58; 1.10]	0.37
Eosinophils, 10^9^/L	0.2 [0.1; 0.3]	0.14 [0.07; 0.21]	0.12
Eosinophils, %	2.2 [1.1; 3.2]	1.7 [1.0; 2.7]	0.20
Platelets, 10^9^/L	220.0 [195.5; 268.0]	210.0 [177.0; 251.3]	0.04

Data are presented as median [25%; 75%].

**Table 3:** MACE odds ratio in patients depending on Lp(a) concentration and blood immune cell distribution according to the median.

	Lp(a) < 30 mg/dL	Lp(a) ≥ 30 mg/dL
Monocytes < 0.54×10^9^/L	1	1.22 (0.53–2.78)
Monocytes ≥ 0.54×10^9^/L	1.0 (0.44–2.28)	2.69 (1.14–6.34) *
LMR ≥ 4.18	1	1.70 (0.75–3.85)
LMR < 4.18	1.88 (0.80–4.41)	3.07 (1.33–7.04) *
Neutrophils < 5.04×10^9^/L	1	1.38 (0.59–3.21)
Neutrophils ≥ 5.04×10^9^/L	0.76 (0.33–1.74)	1.65 (0.96–4.92)
NLR < 2.66	1	1.02 (0.45–2.30)
NLR ≥ 2.66	1.04 (0.45–1.10)	2.61 (1.17–5.82) *

* *p* < 0.05; LMR—lymphocyte-to-monocyte ratio, NLR—neutrophil-to-lymphocyte ratio.

## Appendix A

**Table A1:** Therapy performed in the examined patients.

Therapy	MACE *n* = 121 (%)		without MACE *n* = 79 (%)		*p*-Value
Antiplatelet therapy	92	(76)	65	(82)	0.38
Anticoagulants	31	(26)	18	(23)	0.74
ACE- inhibitors	57	(47)	31	(39)	0.31
Angiotensin II receptor antagonists	36	(30)	25	(32)	0.87
Beta-blockers	107	(88)	70	(89)	1.00
Calcium channel antagonists	39	(32)	24	(30)	0.88
Nitrates	20	(17)	5	(6)	0.04
Diuretics	37	(31)	24	(30)	1.00
